# Exploring tradeoffs among diet quality and environmental impacts in self-selected diets: a population-based study

**DOI:** 10.1007/s00394-024-03366-2

**Published:** 2024-04-07

**Authors:** Rachel Mazac, Matti Hyyrynen, Niina E. Kaartinen, Satu Männistö, Xavier Irz, Kari Hyytiäinen, Hanna L. Tuomisto, Chiara Lombardini

**Affiliations:** 1 Faculty of Agriculture and Forestry, Department of Agricultural Sciences and Helsinki Institute of Sustainability Science, University of Helsinki, Helsinki, Finland; 2 Natural Resource Institute of Finland, Helsinki, Finland; 3 Finnish Institute for Health and Welfare, Helsinki, Finland; 4 Faculty of Agriculture and Forestry, Department of Economics and Management and Helsinki Institute of Sustainability Science, University of Helsinki, Helsinki, Finland; 5 Faculty of Agriculture and Forestry, Department of Agricultural Sciences and Helsinki Institute of Sustainability Science, Natural Resources Institute Finland (Luke), University of Helsinki, Helsinki, Finland

**Keywords:** Dietary patterns, FFQ, Health, Multicriteria analysis, Environmental impacts, Sustainability

## Abstract

**Purpose:**

Proposed sustainable diets often deviate dramatically from currently consumed diets, excluding or drastically reducing entire food groups. Moreover, their environmental sustainability tends to be measured only in terms of greenhouse gases emissions. The aim of this study was to overcome these limitations and identify a cluster of already adopted, relatively healthy diets with substantially lower environmental impacts than the average diet. We also aimed to estimate the reduction in multiple environmental impacts that could be achieved by shifting to this diet cluster and highlight possible tradeoffs among environmental impacts.

**Methods:**

The diet clusters were identified by applying energy-adjusted multiple factor analysis and hierarchical clustering to the dietary data of the National FinHealth 2017 Study (n = 5125) harmonized with life cycle assessment data on food products from Agribalyse 3.0 and Agri-Footprint using nutrient intakes and global warming potential, land use, and eutrophication of marine and freshwater systems as the active variables.

**Results:**

We identified five diet clusters**,** none of which had the highest overall diet quality and lowest impact for all four environmental indicators. One cluster, including twenty percent of the individuals in the sample was identified as a “best compromise” diet with the highest diet quality and the second lowest environmental impacts of all clusters, except for freshwater eutrophication. The cluster did not exclude any food groups, but included more fruits, vegetables, and fish and less of all other animal-source foods than average. Shifting to this cluster diet could raise diet quality while achieving significant reductions in most but not all environmental impacts.

**Conclusion:**

There are tradeoffs among the environmental impacts of diets. Thus, future dietary analyses should consider multiple sustainability indicators simultaneously. Cluster analysis is a useful tool to help design tailored, socio-culturally acceptable dietary transition paths towards high diet quality and lower environmental impact.

**Supplementary Information:**

The online version contains supplementary material available at 10.1007/s00394-024-03366-2.

## Introduction

The transition to sustainable diets is critically important to reduce the negative impacts of food systems on the environment and decrease diet-related non-communicable diseases. All food system activities together, including producing, processing, transport, retail, preparing, consuming, and wasting food, contribute one third of the global greenhouse gas (GHG) emissions [1]. Agriculture is also the leading cause of the Earth system surpassing planetary boundaries in biodiversity loss and nutrient flows [2]. Changes toward sustainable diets are crucial for keeping the earth system within safe planetary boundaries [3].

A healthy diet, however, is not necessarily an environmentally sustainable diet, and tradeoffs among nutritional quality and environmental impacts are not well understood [4]. For example, higher dietary nutritional quality may be associated with lower agricultural land use but not always with less fertilizer, pesticides, or water use depending on how nutritional quality is measured (see e.g., Healthy Eating Index-2015 and the Alternative Healthy Eating Index-2010) [5, 6]. On the other hand, diets with lower environmental impacts (e.g., achieved by restricting total food intake, increasing energy-dense foods, or cutting intakes of all animal-sourced foods) may not meet all the nutritional requirements of a healthy diet [7]. Yet, synergies between reduced environmental impact and increased healthiness have also been identified for diets with lower red and processed meat intakes, as well as higher plant-based food intakes (e.g., legumes), associated with superior health outcomes (i.e., lower risk of morbidity and mortality) [8].

One approach to identify relatively sustainable diets uses *ex-ante* modeling (e.g., optimization, simulations) for comparing current and theoretical diet scenarios [9, 10]. However, these speculative sustainable diets may not be culturally acceptable, which limits the real-world usefulness of such approaches [9, 11]. An alternative approach is to identify sustainable diets that are already adopted in a certain subpopulation since it is more likely that such self-selected diets are more culturally acceptable to the rest of the population [7, 12]. Using this alternative approach, Vieux et al. (2020) identified a tradeoff between lower GHG emissions and higher nutritional quality: the diet cluster with the lowest GHG emissions had also the lowest nutritional quality [7]. A Dutch study showed the existence of tradeoffs amongst different environmental impacts as they could not identify a dietary pattern for which both blue water use and GHG emissions were the lowest [12].

Our study aims to identify healthy diets already adopted by large clusters of Finnish adults (hereafter called self-selected diets), but with a substantially lower environmental impact than the average Finnish diet. Secondly, we explored potential health and environmental tradeoffs in these self-selected diets. We build on the methods of Vieux et al. (2020), extending their framework to include a richer array of indicators reflecting the environmental impacts of dietary choices and updating the findings for the Finnish population [7]. Thirdly, we estimated the potential reduction in environmental impacts that could be achieved if the Finnish adult population would adopt this relatively healthy and less environmentally harmful diet.

## Materials and methods

### Study population

We used data from the National FinHealth 2017 Study conducted by the Finnish Institute for Health and Welfare aiming at producing information on health, health behavior, functional capacity, and wellbeing in Finnish adults [13]. The representative sample (N = 10,247) were invited to a thorough health examination alongside a self-administered health questionnaire. Of the invited, 58% (x = 5952) participated in the examination during which they underwent clinical measurements and were asked to fill in a food frequency questionnaire (FFQ) during the visit or later at home on paper or electronically. The present study comprised participants, who returned FFQs (n = 5302). Exclusion criteria included incompletely filled (n = 110) or duplicate FFQs (paper and electronic n = 9) and consent withdrawals (n = 7). In addition, 0.5% of participants in both ends of the sex-specific daily energy intake distributions were excluded (n = 51) resulting in an analytical sample of 5125 adults [14].

### Sociodemographic and lifestyle factors

Information on participants' sex, age, and residential area were obtained from the sampling frame. Anthropometrics (height (cm), weight (kg), and waist circumference (cm)) were measured during the health examination by trained research staff. Body mass index (BMI) was calculated as weight (kg) divided by squared height (m). Information on sociodemographic (municipality of residency, education, household income) and lifestyle factors (smoking status, leisure-time physical activity) were gathered by a self-administered questionnaire. Municipality of residency was defined by location in three nominal categories (urban, semi-urban, and rural municipality). Based on their answer to the question: "How many years have you studied full-time altogether, including primary school?", participants were divided into educational tertiles—low, medium, and high—by birth year. The general rise in education levels and the related increase in the average number of school years over recent decades in Finland means that this division in tertiles leads to different number of years of schooling among individuals in different birth-cohorts corresponding to the levels low, middle, and high education. Regarding income, participants chose one option from the predefined income groups ranging from below 15,000 to over 90,000€ per year, defined for the household income of the previous year. Smoking status was assessed by questions on smoking history, and current smoking status was categorized according to a four-level scale (Smoking status: has never smoked regularly, has stopped smoking over 1/2 years ago, has stopped smoking less than 1/2 years ago, smokes). Leisure-time physical activity included four categories: low (light activities such as reading and watching television); medium (walking, gardening, or other activities ≥ 4 h/week); high (running, swimming or other physically demanding activities ≥ 3 h/week) or very high (competition or other heavy sports several times/week) [13].

### Dietary intake data

The validated FFQ included 134-items and inquired into the habitual food consumption over the past 12 months [15, 16]. The items were composed of foods, mixed dishes, and beverages commonly used in Finland in 2017 [13, 17]. The consumption of each item was recorded by ten frequency categories ranging from none to 6 or more times a day. Sex-specific^1^ portion sizes were specified for each item (e.g., glass, slice and volume). The National Food Composition Database, Fineli® and the FINESSI software of THL were used to calculate the average daily consumption of foods (ingredient level) and intakes of energy and nutrients [18]. The modified Baltic Sea Dietary Score (mBSDS) was used as an indicator of overall diet quality [19]. The mBSDS measures the adherence to a healthy diet in Northern Europe and includes eight components covering selected food groups and nutrients. These components include consumption of fruits and berries, vegetables, whole grain cereals, low-fat milk, fish, red and processed meat, intake of alcohol (100% ethanol), as well as the ratio of polyunsaturated fatty acids to saturated and trans-fatty acids. The mBSDS is primarily intended for use in epidemiological studies focusing on overall diet quality and health or other food-related phenomena. The score ranges between 0–22 points. Higher points indicate better overall diet quality in relative terms. Therefore, there is no desired threshold value for this score.

## Environmental impact data and dietary impacts

### Impacts and food product source

In this study, we extended the approach by Vieux et al. (2020) from GHG emissions with the addition of impacts land use, freshwater eutrophication, and marine eutrophication [7]. Individual product level life cycle assessment (LCA) data—matched to the foods at the ingredient level, see all foods in **SM **Table 1—was sourced from the Agri-footprint [20] and Agribalyse 3.0 LCA Databases [21] using the OpenLCA 1.10.3 software [22]. We used the characterization factors from the ReCiPe Midpoint (H) method to calculate Global Warming Potential (GWP; additional radiative forcing of all GHG emissions in kg CO_2_ equivalents during 100-year time frame), land use (m^2^ annual crop equivalents), and marine eutrophication (measured in terms of emitted kg N equivalents) and freshwater eutrophication (measured in terms of emitted kg P equivalents) [23]. The system boundaries were from cradle to consumer, including cooking at consumer, the cooking process is described in the **SM **Table 2 with the LCI source data.

Agribalyse is a multi-indicator French Life Cycle Inventory (LCI) Analysis database with data for over 2500 products produced in France [24]. We adjusted the French LCI data to reflect foods as consumed in Finland based on the amount of food imported vs. exported in Finland, with all imports assuming to be from other European countries; the changes are as described below, with detailed information on the process in the SM. If a larger portion of the product was imported into Finland (i.e., with an import to export ratio > 1) based on UN FAO STAT data (FAO STAT), we changed the product inventory data from French average electricity use to electricity use in Europe without Switzerland. If the import ratio was < 1 (e.g., all livestock products and grains to feed livestock) these items were considered ‘produced in Finland’. Then, we adjusted product inventory data of each item from the Agribalyse 3.0 Database to Finnish average electricity use, where products were produced primarily in Finland. Further, the livestock production processes were modified by changing feed to be produced in Finland based on data from the Agri-footprint life cycle inventory database.^2^

### Impact category selection

Updated Finnish LCI data was used to assess the environmental impacts per kg of food item. Although the Agribalyse 3.0 would have allowed us to consider a wider range of environmental impacts, we decided to focus on these four (GWP, land use, freshwater eutrophication, and marine eutrophication) not only for their relative importance in terms of overall environmental impacts but also as they summarize the key information about the overall impacts, indicated by the following Principal Component Analysis (PCA). We selected these environmental impact categories as they are those which are most studied, and thus, readily comparable in regard to the environmental impacts of food [3, 25]. We also ran a PCA with all 16 of the environmental impact categories generated as a result of the ReCiPe Midpoint (H) assessment method. This PCA showed that 62% of total variation in the impact data could be explained by the first dimension, of which GWP, land use, and marine eutrophication were highly significantly correlated, and an additional 15% (77% total) is explained in the second dimension, most highly correlated with freshwater eutrophication.^3^ The linear regression on all impacts correlated with all others, shows that most of the individual impacts are strongly, positively correlated (*p* > 0.05) with each other.

### Diet impact calculations

To calculate the environmental impacts for each participant in the FinHealth 2017 Study, we matched the LCA data with the data of FinHealth 2017 Study food groups (n = 25 groups) at the ingredient level (n = 81 foods).^4^ We associated each environmental impact with the food ingredients by multiplying the amount consumed (g/person/day) by the impact (unit impact/g product).

### Multiple factor analysis and hierarchical clustering

Following the methodological approach used in Vieux et al. (2020), the statistical analysis comprised two steps [7]. First, we run a multiple factor analysis (also multifactorial analysis) [26–28], hereafter MFA, to the individual diets of the FinHealth 2017 Study participants. The MFA allowed us to summarize the variability of the nutrient intakes and environmental impacts data through a limited number of factors (dimensions). These factors were then used in the second step to identify the diet clusters by agglomerative hierarchical clustering based on the Euclidean distance between individuals [29]. For the MFA, two groups of active variables and six groups of supplementary variables were used. The two active variables groups were: nutrients variables group—46 variables—and environmental impact variables group—4 variables. The five groups of supplementary variables were: intake of foods,^5^ diet quality indicators (mBSDS),^6^ socioeconomic variables (age, gender, education, household income and municipality of residence), health and lifestyle variables (BMI, waist circumference, smoking status, leisure-time physical activity), total energy intake as well as importance of plant proteins in the diet measured by the intake of plant-based proteins over total proteins and by the ratio of plant proteins to animal proteins.^7^

Before running the MFA, we made some adjustments to the data. First, we adjusted all food and nutrient intakes as well as the environmental impact variables for total energy intake using the residual method [30, 31] as done in Vieux et al. (2020) [7]. This energy adjustment method was done to focus on the variability in the dietary composition among individuals independent of energy intake [31]. Second, to get rid of measurement scales, we scaled to the standard deviation of all variables adjusted for total energy intake. Third, we applied a weighting factor to correct for the effects of non-response in the survey data and ensure that the results of our analysis can be generalized to the entire Finnish adult population.

Both MFA and hierarchical clustering were performed using R Statistical Software [32] with the packages FactoMineR [33] and Factoshiny [34]. The number of clusters was chosen based on how much inertia decreases as the number of clusters increase and on the interpretability of clusters*.* Inertia refers to the total within-cluster sum of squares, which measures the compactness of the clusters. It's a measure of how internally coherent the clusters are: the lower the inertia is, the more compact is the cluster. To increase the robustness of the clusters, we used k-means consolidation [35]. All statistical significance tests use a 5% level of significance.

To assess if the cluster averages are significantly different from the sample averages, we estimate the v-test statistics for each variable and for each cluster. If the v-test statistics is positive, then the variable has a higher average value in the cluster compared to the whole sample. If the v-test value is negative, then the variable has a lower average value in the cluster compared to the whole sample. The higher the absolute value of the v-test statistic the stronger the characterization of that cluster by that variable is. The significant values of the v-tests are reported in **SM **Table 15–19.

We conducted post-hoc comparisons between the clusters. The Levene’s test (see **SM **Table 11) indicated the presence of heteroscedasticity, which rules out the use of the ANOVA and Tukey tests. The Shapiro–Wilk test (see **SM **Table 12) suggests that the variables in the clusters are not normally distributed, which excludes the use of Welch-ANOVA and Games-Howell tests. We thus run the non-parametric Kruskal–Wallis and Dunn tests to conduct the post-hoc comparisons to determine if the distributions of the variables differ between clusters. The results of these two tests are reported in the **SM **Table 13 and 14. For non-ordinal, categorical variables, the post-hoc comparisons were done by first running a Chi-square test of independence. Since the Chi-square results were significant, we tested for pairwise differences in proportions across clusters by calculating the maximum differences in proportions for each pair of clusters and comparing these differences against a critical value derived from the Chi-square distribution, adjusted for multiple comparisons using the Bonferroni correction.

### Environmental impact of dietary transition

We also calculated the change in environmental impacts of the dietary transition scenario were all individuals aged 18 or above in Finland to adopt the Good compromise cluster diet.^8^ For each environmental impact the percentage change in that impact is calculated comparing the average environmental impact of the Good compromise cluster with the average environmental impact of the whole sample average.

## Results

In the MFA, the first dimension accounted for 40% of the variability, the first two dimensions together for 55%. Three of the four environmental impacts are directly correlated with each other, namely land use, GWP, and marine eutrophication, while freshwater eutrophication is not (Supplementary Fig. 3); freshwater eutrophication was not found to be correlated to a majority of the other environmental impact categories^9^. Beef and pork consumption are positively correlated with GWP while the ratio of plant proteins to animal proteins is negatively correlated with GWP.^10^ Additional correlations of note include sucrose (sucs) and alcohol (alc) intakes which are inversely correlated with most other nutrient intakes (see the correlation circle for active variables in Supplementary Fig. 3). Socioeconomic, demographic, and health/lifestyle variables are not strongly correlated with the other variables.

**Fig. 1:**
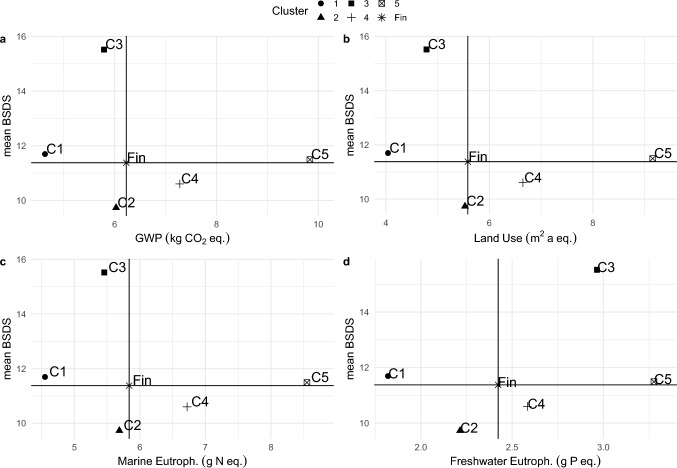
The five diet clusters (C1-5) and their relation to average Finnish diet (Fin) in terms of mean modified Baltic Sea Diet Score (mBSDS) and environmental impacts: **a**. Global Warming Potential (GWP in kg CO2 eq.), **b**. Land Use (m2 annual cropland eq.), **c**. marine eutrophication (kg N eq.), and d. freshwater eutrophication (kg P eq.), respectively

Five clusters were retained in the hierarchical clustering following the inertia gain and for interpretability of clusters as in Vieux et al. (2020) [7]. No cluster had both the highest diet quality and the lowest environmental impacts (Table 1 and Fig. 1). Cluster 1 had the lowest environmental impacts but a mBSDS slightly higher than the sample mean while cluster 3 had the highest mBSDS but only the second lowest value of GWP, land use and marine eutrophication. Cluster 3 stands out from the others in terms of consistently lower environmental impacts with the highest diet quality, a pattern seen particularly in Fig. 1a, b, c. Moreover, cluster 3 had the second highest negative impact on freshwater eutrophication. The cluster 3 diet was identified as a good compromise between relatively high dietary quality and relatively low environmental impacts.^11^ The next two sections focus on the characterization of clusters 1, 3, and 5. We name these clusters, respectively: Lowest impact, Good compromise, and Highest impact, where impact refers to the four negative environmental impacts considered.

**Table 1:** Clusters characterized by diet quality and environmental impacts of food items consumed in a day

Variable	All	Cluster 1 Lowest impact	Cluster 2	Cluster 3 "Good compromise"	Cluster 4	Cluster 5 Highest impact	Dunn-test^a^, p < 0.05
n	5125	799	1953	936	1216	221	
%	100	16	38	18	24	4	
Diet quality							
Baltic Sea Diet Score (mBSDS)^b^	11.38	11.70*	9.73*	15.52*	10.60*	11.49	All significant but 1–5
Environmental Impact							
Global Warming Potential (kg CO_2_-eq/day)	6.23	4.63*	6.03*	5.79*	7.28*	9.83*	All significant
Land Use (m^2^ annual cropland-eq./day)	5.58	4.04*	5.53*	4.78*	6.65*	9.16*	All significant
Marine Eutrophication (g Nitrogen-eq./day)	5.84	4.6*	5.7*	5.5*	6.7*	8.6*	All significant
Freshwater Eutrophication (g Phosphorus-eq./day)	2.42	1.8*	2.2*	3.0*	2.6*	3.3*	All significant

^a^The Dunn test is used to compare the differences in pair-wise group medians in a post-hoc manner following a Kruskal–Wallis test when the assumptions for the Tukey and ANOVA tests are not met, that is, normally distributed data and homoscedasticity. For instance, All significant but 1–5 means that all pairwise comparisons indicate that there are statistically significant apart from the pairwise comparison 1–5

^b^The Baltic Sea Diet Score is a general a priori diet quality index developed to depict a healthy Nordic diet and composed of foods that are typically grown and consumed in the Nordic countries [19, 36]. The score ranges between 0–22 points. Higher points indicate better overall diet quality in relative terms. Therefore, the score has no desired threshold value

^*^ The cluster mean is significantly different from the sample mean based on v-test statistics. All significant V-test values are reported by cluster in **SM **Table 15–19

As shown in Table 1, Cluster 3 had the highest mBSDS, 15.52 compared to the sample average of 11.38, which however is still far from the maximum value of 22 points.

**Fig. 2:**
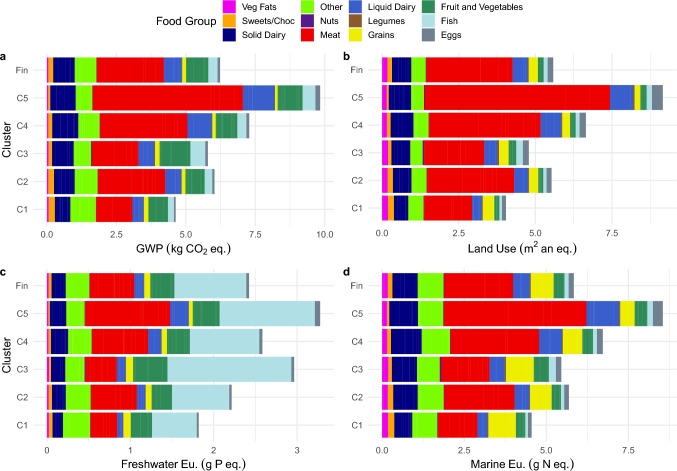
Environmental impacts of **a**. global warming potential (GWP), **b**. land use, **c**. freshwater eutrophication (Eu.), **d**. and marine eutrophication (Eu.) and the contribution to each impact per day of each food group by cluster (C1-5) and the average of the sample (Fin). NOTE: Meat includes beef, pork, poultry, offal, processed meats, and lamb/mutton; Other includes beverages (alcoholic and non-alcoholic), edible fats, and condiments

Individuals in the Good compromise cluster had the second highest ratio of plant to animal proteins after the Lowest impact cluster while the Highest impact cluster had the lowest ratio (Table 2). The Good compromise cluster had the highest intake of fruit and vegetables, legumes, nuts, and fish while it had the second lowest intake of red and processed meat. No food groups were absent from the Good compromise cluster diet. Individuals in the Highest impact cluster had the highest consumption of red and processed meat, dairy products (liquid and solid), and eggs, the second highest fish consumption and the second highest vegetable consumption. The Lowest Impact cluster had the lowest intake of red and processed meat, eggs, and fish. Animal source foods contribute the most to all environmental impacts across all clusters (Fig. 2). The Meats and Dairy food groups contributed the most to GWP, land use, and marine eutrophication in all clusters, where the Fish and Meats contributed the most to freshwater eutrophication. The relative impacts of most other food groups are limited across impact category and cluster.

**Table 2:** Selected dietary characteristics of the Lowest impact, Good compromise, and Highest impact clusters

Variable	Sample Average	Cluster 1 Lowest impact	Cluster 3 Good compromise	Cluster 5 Highest impact	Dunn test^c^ at p = 0.05 significance level
Nutrients					
Total Energy (kJ/day)	8693	9860*	8970*	11,500*	All significant
Carbohydrate (E%)	40	45*	55*	32*	All significant
Fat (E%)^a^	42	31	34	31	All significant but 1–5
Saturated fat (E%)^a^	16	13	13	12	All significant but 1–3
Protein (E%)^a^	21	13	18	20	All significant but 4–5
Protein (g/day)	96	74*	98*	141*	All significant
Plant/animal protein ratio	0.40	0.67*	0.48*	0.19	All significant
Food groups (g/day)					
Fruit and vegetables^a^	441	387	646	506	All significant but 1–2, 4–5
Legumes^a^	2	3	5	2	All significant
Nuts and seeds	7	6	13*	7	All significant but 2–4,1–5, 2–5,4–5
Grains^a^	127	155	140	78	All significant
Pork^b^	32	18 *	23*	71*	All significant
Beef^b^	29	14*	19*	78*	All significant
Processed meat^a,^^b^	40	30	26	45	All significant but 1–3, 2–4
Poultry^b^	40	20*	39*	88*	All significant
Fish	37	23*	64*	49*	All significant but 4–5
Eggs	35	25*	37	67*	All significant but 2–3
Liquid dairy products^a^	446	291	403	572	All significant but 2–3
Solid dairy products^a^	62	57	55	73	All significant but 1–3, 2–4, 1–5, 2–5, 4–5
Vegetable-based fat^a^	20	20	26	18	All significant but 1–2, 1–4, 2–4, 1–5, 2–5, 4–5
Sweets and chocolate^a^	26	36	21	13	All significant but 3–4

The foods which comprise the food groups are included in the R-Script employed when assigning food groups, see Code Availability; percent of total energy intake (E%)

^a^V-test not run, significant differences determined by only posthoc Kruskal–Wallis and Dunn’s Test

^b^In Meat food group with game, lamb/mutton, and offal

^c^The Dunn test is used to compare the differences in pair-wise group medians in a post-hoc manner following a Kruskal–Wallis test when the assumptions for the Tukey and ANOVA tests are not met, that is, normally distributed data and homoscedasticity. For instance, All significant but 1–5 means that all pairwise comparisons indicate that there are statistically significant apart from the pairwise comparison 1–5

^*^Cluster mean is significantly different from the sample mean based on v-test statistics

If the Finnish adult population were to adopt the diet of the Good compromise cluster and this would directly lead to equivalent changes in Finnish agriculture, such a shift would reduce the contribution of Finnish food system on GWP by 7% (− 0.72 Mt CO2eq/year), land use—occupation, use, transformation of all land use types—by 14.3% (1.31 million of km^2^ annual crop eq/year) and marine eutrophication by 6.5% (626.47 tNeq/year). The freshwater eutrophication measure, however, would increase by 22.3% (891.66 tPeq/year). This increase occurs as the Good compromise diet has on average higher freshwater eutrophication due to the much higher average intake of fish (64 g/d) compared to the average of the whole sample (37 g/day).

The adoption of the Good compromise cluster diet of the entire adult Finnish population would increase the average intake of fruit and vegetables (75 kg/per capita/year), fish and fish products (73.3 and 63.98 kg/per capita/year respectively), and legumes (98.14 kg/per capita/year), while there would be a decrease in the consumption of beef (34.94 kg/per capita/year) processed meat intake (35.63 kg/per capita/year), pork (28.72 kg/per capita/year) and poultry (3.3 kg/per capita/year).^12^

The Good Compromise cluster had older individuals and more women compared to the sample average, the highest level of education of all clusters, and an average income that was not statistically different from the sample average (~ 50 000 € before tax) (Table 3). Their BMI was lower than the average in the sample but not statistically different from that of clusters 1 and 2. The Good Compromise cluster’s individuals were also more physically active in their leisure-time than the sample average at par with the Highest impact and second highest impact clusters and had the least current smokers and ex-smokers of all clusters. Compared to the sample mean, the individuals in the Highest Impact cluster were younger (49 years old) and predominantly males (35% women), had higher average income together with cluster 4, but had the same average level of education as the whole sample. In the Lowest Impact cluster, there were more women, the average level of income was lower and the age higher than in the sample, while the level of education did not differ statistically from sample averages.^13^ Only pairwise comparisons 1–3 and 2–3 were significant for the non-ordinal, categorical variable Municipality (see Table 3 for description).

**Table 3:** Characterization of the individuals belonging to the Lowest impact, Good compromise, and Highest impact clusters

Variable	All	Cluster 1 Lowest impact	Cluster 2 Average impact	Cluster 3 "Good compromise"	Cluster 4 Second highest impact	Cluster 5 Highest impact	Dunn test^e^ at p = 0.05 significance level
N	5125	799	1953	936	1216	221	
%	100	16	38	18	24	4	
Sociodemographic variables							
Average age (years)	55.88	60.97*	54.85	61.44*	51.22*	48.64*	All significant but 1–3 and 4–5
Sex (1 = male, 2 = female)	1.56	1.53	1.58	1.66	1.51	1.35*	All significant but 1–2 and1-4
Women (%)	56.10	52.69	58.47	65.92	50.82	34.84	
Living in urban municipalities^a^ (%)	67.34	64.8	65,00	72.1	69.00	67.4	
Education^b^	2.04	2.05	1.98*	2.25*	1.96*	1.99	Only 1–3, 2–3,4–3, 5–3 significant
Education (% in category 1—low education)	31.00	30.66	33.69	21.69	34.05	31.22	
Household income^c^	5.02	4.51*	4.95*	4.98	5.37*	5.80*	All significant but 2–3,and 4–5
Health indicators							
Current smoking status^d^	1.72	1.69	1.77*	1.48*	1.81*	1.77	Only 1–3,2–3,4–3, 5–3 significant
Never smoked (%)	58.40	59.07	57.19	65.17	55.18	55.66	
Body Mass Index (kg/m2)	27.41	26.65*	27.38	26.96*	28.21*	27.95	All significant but 1–3,2–3,2–5, 4–5
Leisure time physical activity^f^	2.06	1.93*	1.97*	2.21*	2.14*	2.24*	All significant but 1–2,3–4,3–5,4–5
Percentage 'Low' leisure time physical activity level (PAL = 1)	24.23	29.41	28.21	14.42	22.53	21.27	

^a^**1 Urban municipalities**: Urban municipalities are those municipalities in which at least 90 per cent of the population lives in urban settlements, or in which the population of the largest urban settlement is at least 15,000. **2 Semi urban municipalities**: Semi-urban municipalities are municipalities in which at least 60 per cent but less than 90 per cent of the population lives in urban settlements, and in which the population of the largest urban settlement is at least 4,000 but less than 15,000. **3 Rural municipalities**: Rural municipalities are those municipalities in which less than 60 per cent of the population lives in urban settlements, and in which the population of the largest urban settlement is less than 15,000, as well as those municipalities in which at least 60 per cent but less than 90 per cent of the population lives in urban settlements, and in which the population of the largest urban settlement is less than 4,000

^b^Education: 1 = low, 2 = medium, 3 = high

^c^Previous year's household income: 1 = below 15,000€, 2 = 15,001–25000€, 3 = 25,001–35000€, 4 = 35,001–45000€, 5 = 45,001–50000€, 6 = 50,001–60000€, 7 = 60,001–70000€, 8 = 70,001–80000€, 9 = 80,001–90000€, 1 0 = above 90,000€

^d^Smoking status: 1 = has never smoked regularly, 2 = has stopped smoking over 1/2 years ago, 3 = has stopped smoking less than 1/2 years ago, 4 = smokes

^f^Leisure time physical activity: 1 = low, 2 = medium, 3 = high, 4 = very high

^e^The Dunn test is used to compare the differences in pair-wise group medians in a post-hoc manner following a Kruskal–Wallis test as the assumptions for the Tukey and ANOVA tests, normally distributed data and homoscedasticity, are not met. In the last column: All significant but 1–5 indicates that all pairwise comparisons are statistically significant apart from the pairwise comparison 1–5. For ease of reporting, when the majority of pairwise comparisons is not significant, only the significant pairs are indicated as in Only 1–3,2–3,4–3, 5–3 significant

### Regression and principal components analysis on environmental impacts correlations

The linear regression on all impacts correlated with all others, shows that most of the individual impacts are strongly, positively correlated (*p* > 0.05) with each other. Human carcinogenic toxicity is not significantly correlated with freshwater eutrophication nor with global warming potential. Human noncarcinogenic toxicity is not significantly correlated with human carcinogenic toxicity, nor is human carcinogenic toxicity significantly correlated with ionizing radiation, land use, marine eutrophication, stratospheric ozone depletion, and terrestrial acidity. Otherwise, all other environmental impacts are significantly, positively correlated with all others.

Principal Components Analysis (PCA) shows, similarly, that all impacts vary in the same direction (Supplementary Fig. 1). In the PCA, 62.35% of the variance is explained by the first dimension and an additional 14.77% by the second dimension. After two dimensions, 77.12% of the variance is explained and 85.46% after the third dimension (**SM **Table 8). Global warming potential is well expressed in the first dimension and correlates with land use and marine eutrophication, where freshwater eutrophication is less well projected in the first to dimensions (Supplementary Fig. 2). It is for these reasons, as well as for comparison of impacts of whole diets with other studies, that we chose to use global warming potential, land use, and marine and freshwater eutrophication, since with these four, we can account of over 67% of the total variance.

## Discussion

### Self-selected diets and tradeoffs among environmental impacts

We identified a relatively healthy diet with a substantially lower environmental impact than the average Finnish diet taking into account a wider range of environmental impacts than previously examined in the literature. This diet did not exclude any food group and was already adopted by 18% of Finnish adults, though a majority of these were older, highly educated women. In terms of dietary quality, even though the Good compromise diet represents a significant improvement compared to the diet of an average Finn [17, 37], it still falls short of the Finnish nutritional recommendations [38]. The dietary challenges of Finland are evident in all clusters. For example, even though the Good Compromise cluster had the lowest BMI, this was, however, in the overweight range for adults, 25–30 kg/m^2^ [39]. Similarly, even though the adoption of the Good compromise cluster diet would significantly reduce three of four investigated environmental impacts, this diet is still is far away from the EAT-Lancet diet, a global recommended “planetary health” diet which takes into account both human health and environmental impacts [3]. Compared to the EAT-Lancet diet, the Good compromise diet has a much higher consumption of beef (+ 185%), pork (+ 239%), eggs (+ 196%), fish (+ 139%), and tubers and starchy vegetables (+ 95%), a somewhat higher consumption of fruits (+ 34%) and vegetables (+ 35%) and poultry (+ 39%), and a lower consumption of dairy (-15%), legumes (-93% not including peanuts) and whole grains (-37%). It must however be underlined that the Good compromise diet is meant to describe an already adopted diet that is healthier and has lower environmental impacts than the average diet of a Finnish adult: it is a self-selected diet that emerges from the cluster analysis, not a prescriptive diet as the ones from the national dietary recommendations or the EAT-Lancet diet. Thus, the comparison with recommended diets is meant to show how even this Good compromise diet is still far from ideal, as are current Finnish adult diets, with the average diet far from both the Nordic Nutrition Recommendations and the EAT-Lancet diet.

Our study not only confirms previous results that a self-selected diet with the highest diet quality is not always the one with the lowest GHG emissions [7, 40], but also shows that the result holds for a wider set of environmental impacts than previously considered in the literature. Further, while in Vieux et al. (2020) the lowest GHG emissions were associated with the cluster with the lowest dietary quality, in our analysis the cluster with the lowest GWP (Lowest impact cluster) had an average diet quality slightly higher from the sample average [7]. This suggests a weaker tradeoff between climate impact and diet quality at least in Finland. The Good compromise cluster and the “More Sustainable” cluster in [7] have a similar overall percentage of the total population (18.3 and 18.0%, respectively) and both had more women (61.4 and 62.0%, respectively). The Good compromise cluster had nearly twice the amount of fruits, vegetables, and livestock meat compared to the More Sustainable cluster. This greater amount of livestock meat, the higher average caloric intake (2152 and 1939 kcal, respectively) and the narrower system boundaries in Vieux et al. (2020) LCI is likely to explain the higher average greenhouse gases emissions of the Good compromise cluster diet than the More Sustainable (5.79 kg CO2-eq/day and 3.8 kg CO2-eq/day, respectively).

The analysis also highlights the existence of tradeoffs among environmental impacts. We found a tradeoff between freshwater eutrophication and the other three environmental impacts of global warming potential, land use, and marine eutrophication. These three impacts correlated with each other, suggesting that if the diet increases one of the environmental impacts, it also increases the two others [41]. Lack of correlation of freshwater eutrophication with the other impacts in the Good compromise diet is due to the higher intake of fish. The fish in these diets is represented using the environmental impact data for cultivated salmon trout requiring feed input, and we found that the consumption of this fish contributes to freshwater eutrophication impacts more than other food groups. It is known that freshwater fisheries cause eutrophication [42], and eutrophication is identified as a challenge for other European diets [43]. Thus, although the Good compromise diet is associated with lower environment impacts for GWP, land use, and marine eutrophication compared to the other diets, it is associated with higher freshwater eutrophication than the other cluster diets.

To put the reductions in environmental impact from the adoption of the Good compromise cluster diet in perspective, according to Statistics Finland's data, the total GHG emissions in Finland in 2020 amounted to 47.8 Mt CO2eq [44]. Thus, a change in dietary composition of current diets to the Good compromise diet would reduce total Finnish GHG emissions by roughly 1.5% and GHG emissions from the current diets by 7% (see **SM **Table 6). Diet-related land use would decrease by 14.3%. In comparison with other dietary transitions, the adoption of vegan diets in Finland would see ~ 15% reduction in carbon emissions and ~ 50% reduction in land use in compared to current diets [45, 46]. Such impact reductions seen in our results are significant considering that the diet represented by the Good compromise cluster does not require to exclude any food group, unlike vegan diets.

Due to the tradeoffs within environmental impacts, however, not all environmental impacts would decrease. Freshwater eutrophication would increase by 22.3% due to high fish consumption in the Good compromise diet compared to the other cluster diets. Given that agriculture and crop cultivation are the cause of 47% of phosphorus loads [47], adoption of the Good compromise diet would lead to a 10% increase in total phosphorus loads. However, the overall societal impact due to the environmental and health impacts of the adoption of the Good compromise diet depends on the relative valuation of these impacts. In practice, most cost–benefit studies of sustainable dietary shifts have found that health benefits tend to outweigh environmental costs or benefits [48].^14^

Our results confirm that the quantity and nature of dietary protein are key determinants of environmental impact. Diets containing less protein overall, and relatively more plant-based protein, have a lower impact, although they are not necessarily healthier [40, 49]. Such results can be seen in Table 2. Despite the Good compromise cluster having the same amount of total protein intake as the sample average, the Lowest impact and Good compromise clusters have the highest and second highest plant to animal protein ratio while the Highest impact cluster has the lowest plant to animal protein ratio. Our findings are thus in alignment with a number of other studies which describe the relatively lower impacts of diets with higher plant to animal protein ratios and urge a shift toward more plant-based protein intake in lieu of animal source protein [7, 50]. Additionally, protein intake is not the main nutrient of concern nor the only nutritional unit which is useful for comparison of dietary healthiness and environmental impacts. Micronutrients and vitamins, as well as protein quality play an important role in diets which cannot be neglected in sustainable diet discussions [51, 52]. Nevertheless, for some population groups—particularly male, high-income, urban and semi-urban dwellers—the main emphasis should be placed on reducing protein intake as there is evidence that a significant share of the population’s protein intake is in excess of dietary recommendations [53], although this may be behaviorally difficult [54, 55].

### Self-selected diets and insights into sustainable dietary transitions

This study shows that a population-wide shift to a self-selected diet, that is a relatively good compromise between higher diet quality and lower environmental impact, would achieve significant reductions in multiple but not all environmental impacts without having to eliminate any food groups, in line with previous literature regarding GHG emissions, land use, and eutrophication [7, 45, 46, 56]. Nevertheless, it would require a shift from animal to plant proteins, with major reductions in red and processed meat intake—by -36% for processed meat and -35% for beef compared to the average of the sample—especially by some individuals with eating patterns farthest from the Good compromise diet. Significant decreases—at least 20% reduction—in animal-sourced food intakes can lead to significant—corresponding 25%—reductions in environmental impacts while still meeting the requirements of healthy diets in Europe [10] and worldwide [3]. Intake of vegetables and fruit and legumes would also have to increase significantly—at least 30 and 50%, respectively—in average diets compared to the Good compromise diet.

Individuals in the Good compromise diet were the oldest, most educated, had the largest proportion of women and healthier lifestyles both in terms of smoking habits and physical activity. This result is in line with previous results indicating that that the higher educated, those that live urban areas, and women adhered more closely to recommended food consumption than others [53]. The Highest impact cluster had more males with a lower level of education than the sample average. Earlier studies also found that consumers of red and processed meat are more likely to be male and less educated [57]. The fact that the average household income in the Good compromise cluster did not differ from the sample average suggests that affordability is not likely to be a key obstacle in the dietary transition, a result aligned with previous studies in Finland [57] but in contrast with previous studies showing that affordability is often a limiting feature of more nutritious diets with trade-offs in diet quality and diversity [40, 58]. However, there is also evidence that diets rich in plant-based proteins are associated with significantly higher Healthy Eating Index (2010) with a minimal increase in cost [59].

Although the Good compromise diet is adopted by 18% of the sample, making it potentially socio-culturally acceptable, it is nevertheless quite different from some of the other cluster diets. Thus, a shift to this Good compromise diet would require quite significant changes in food consumption by some individuals. Erkkola et al. (2022) indicate that dietary transitions for such high red meat intake consumer profiles would be most effectively achieved through a step-by-step transition from red meat to poultry, then fish, and lastly to plant-based proteins. More generally, even when self-selected and hence relatively socio-culturally acceptable diets are identified, tailored dietary transition paths should be designed based on a good understanding of the diet clusters existing in society. Similar pathways for meat reduction in “consumption corridors” has been suggested in the case of meat for defining “sustainable meat consumption” in the European Union [60, 61]

Our article contributes to a better understanding of sustainable dietary transitions in Finland, and to other Nordic, and European or high-income settings. This analysis allows for a detailed characterization of effective strategies to encourage dietary shifts towards these more sustainable diets. These strategies could be targeted to specific groups based on food consumption patterns and selected socioeconomic, lifestyle, and health characteristics. Given that some clusters differ more from the Good compromise cluster than others in terms of socioeconomics or lifestyle characteristics, there may be some low hanging fruit for tailored, culturally acceptable interventions. For instance, in the cluster with the highest red meat intake and proportion of proteins from animal sources (Highest impact cluster), individuals also had the highest proportion of high leisure time physical activity and of men. Thus, interventions could focus on promoting the substitution of red and processed meats with poultry as a first step towards a sustainable diet, as opposed to suggesting the reduction of animal sourced proteins [57]. As a second example, fish consumption is a cause of considerable freshwater eutrophication impact in the Good compromise cluster. However, high fish consumption is also a main reason why this cluster scores highly in the mBSDS. Therefore, the tailored intervention here would not be to reduce fish consumption per se, but to encourage consumers to transition to plant-based diets—as recommended in the 2023 Nordic Nutrition Recommendations [62]—and substitute more impactful fish from aquaculture and overfished wild-stocks with wild-caught, significantly less impactful, local fish like roach. It is estimated that roach has 2–5 GWP kgCO_2_eq per kg of protein compared to wild fish (5–70) and fish from aquaculture (4–75). Moreover, removing roach fish indirectly reduces eutrophication [63]. Finally, roach does not suffer from overfishing while often wild-caught fish is unsustainably fished. For instance, only 64.6% of marine fishery stock was within biologically sustainable levels in 2019 according to a FAO’s assessment [64].

## Limitations

The LCA database used in the analysis was developed in France and adjusted to reflect Finnish production conditions. This adjustment presents some limitations. Since this database was adapted, further validation of the results is required. Firstly, we assumed all imports to Finland were from other European countries for changes to the LCI data. However, according to the Finnish Environment Institute just over 86% of food imports in Finland are from EU member or other European countries [65]. In Finland, 50% of GHG emissions from agriculture are caused by cultivation of peat soils [44], and those emissions are not considered in this study. If the dietary change resulted in reduction of beef and milk consumption, and those peat soils could be taken away from agriculture, the GHG emission reduction could be even higher. Nevertheless, the order of magnitude of the average GHG emissions associated with the diet appears relatively robust. We estimated that the average annual per capita carbon footprint on the FinHealth 2017 study is 6.23 kg CO_2_eq. /cap./ day based on the energy adjusted and population weighted food intakes. Our results were in line with the values in previous literature, being very close to other assessment methods calculating impacts from the FinDiet2017 intake data at around 6.2 kg CO_2_/cap/day for the current diet [46], within the range of other European diet cluster studies (3.5–7.0 kg CO_2_/cap./day) [7], and in line with food accounting for nearly a quarter of carbon impact from commodity consumption in Finland [66]. In terms of land use, our results are, on average, in a similar range as previously modeled diets in Finland. Our results show the average Finnish diet uses 0.20 ha/cap./year (5.58 m^2^/cap/day). Previous Finnish diet models—modeled with average intake data on localized agricultural production assumptions—totaled 0.33 ha/cap./year, while the other modeled diet options ranged from diets which met current diet recommendations, mixed diets without pork and poultry, to vegetarian diets, with 0.26, 0.19, and 0.17 ha/cap./year in land use, respectively [67]. Additionally, a recent estimate of the land use for the current, average diet in Finland based on the national food consumption survey data (FinDiet 2017) but different impact assessment methods found the current Finnish diet to use around 6.75 m^2^/cap./day [45]. In terms of eutrophication potential, we are also in the range of previously modeled Finnish diets—composed of three meals—who carried out an LCA of school lunches [56]. The Finnish school lunches, both home-made and ready-to-eat, ranged from 0.7 to 4.6 g PO4eq./meal [56]—that is 2.1 to 13.8 g PO4 eq./cap./day or 0.72 to 4.70 g P—if that meal is consumed thrice per day. Our diets ranged from 1.8 to 3.3 g P eq./cap./day, within the same range as the previous estimates, even given that they were meals for children, and thus had lower total energy content.

Life cycle analysis of food production and subsequent consumption in human diets is inherently complex. Though capturing such complexity is attempted in LCA methods, there are important limitations in these methods. Decisions made along the LCA process such as the system boundaries, geographic limits, time scale of impacts, and inclusion or exclusion of impact categories influence the results of studies which apply individual LCA data to whole diets. The scale at which environmental analyses are conducted, whether regional or global, significantly influences the outcomes of sustainability assessments. Particularly evident in LCA, are the regional coefficients and weighting factors which play a pivotal role in shaping impact assessments. These factors hinge on geographic considerations, such as resource availability, location, density, and transportability. The choice of geographic scale, whether it be at the farm, city, national, regional, or global level, is integral in LCA studies and data sourcing. Though food systems and production methods differ greatly across scales, Finland and France are similar across several agri-environmental indicators such as fertilizer application rates (P and N), antimicrobial sales per head of livestock, sale of herbicides, and livestock density [68–71].

The source and geographic location of energy production significantly influence results of LCA. Energy production methods vary across Europe, affecting GWP, with, for instance, France relying heavily on nuclear power while other European regions use solid fossil fuels, oil, petroleum, or natural gas [72, 73]. Lastly, the choice of impact assessment method, such as the commonly used ReCiPe 2016 Midpoint (H) method, has implications for comparing environmental impacts and identifying tradeoffs among impact categories in LCA. These results presented here can be extended to evaluate impact pathways and outcomes in areas like ecosystem damage, human health, and resource use, though they may require breaking down contributions from individual environmental impacts when examining specific effects [23]. It is worth noting that conventional LCAs typically utilize mass as the functional unit, potentially limiting their capacity to consider the primary function of foods, which is to provide essential nutrients to consumers [74, 75].

Our analysis was based on a nationally representative sample of Finnish adults with extensive background data and comprehensive dietary data from a validated FFQ. It should be acknowledged, however, that the FFQ provides relative consumption and intake measures which may not reflect absolute long-term food consumption and nutrient intakes of the Finnish adult population. This data is based on self-reported food consumption and thus might suffer from recall and social desirability bias. Yet, we have considered the nature of our dietary data in the analytical choices and in the interpretation of our results. To improve the generalizability of our results to the general adult population in Finland, we applied survey weights to address possible non-response bias [76].

## Further studies

Future studies could extend these results to reveal more nuanced findings. Further exploration of the sociodemographic variables and their relation to different diet clusters could inform more targeted interventions for diet shifts in more differentiated subpopulations. Addition of data on the costs of the food items that comprise these diet clusters could reveal the financial costs or savings associated with each cluster diet and the adoption of the Good compromise diet. For example, adding purchase price data from local retail stores in Finland and associating those with sociodemographic and environmental impacts can reveal a more complete picture of the sustainability of different diet clusters. Deeper exploration of the diet shifts, which target the ‘low hanging fruit’ of the least taste-cost recommendations, to which is associated the least loss of utility (e.g., palatability, convenience) given the increased healthiness and lower negative environmental impacts [77], can illuminate the first and easiest steps to enact required dietary changes [48].

Our analysis used energy-adjusted values of environmental impacts, food consumption and nutrients intakes. Thus, the estimated potential changes in impacts do not take into account the impact of differences in caloric intake between the clusters but only of changes in their dietary composition. An interesting topic for further study would be to compare the potential reduction in environmental impacts from changing dietary composition to those achievable from reducing excess energy intake [78, 79]. In our data, the average BMI of the sample as well of that of each cluster was in the overweight range, suggesting a significant potential for such reduction [80].

## Conclusions

We found that there are currently consumed diets in Finland, which offer a “Good compromise” between health and environmental outcomes. Healthier lifestyles, higher education, and identifying as a female is associated with the intake of the “Good compromise” diet. No food groups would need to be removed from average diets in Finland, but significant increases in fruits and vegetables, fish, and legumes with reductions in red and processed meat consumption would be required to improve diet quality and reduce certain environmental impacts, with possible tradeoffs amongst environmental impact categories. This study addressed current limitations in diet modeling which often rely on *ex-ante* methods and hypothetical diet models. We expand on previous methods here by identifying self-selected diet clusters which are currently consumed and extended current methods by adding infrequently studied environmental impact categories. Such “real world” diets provide evidence for dietary shift recommendations. The diet clusters identified here can guide eaters and policymakers in making stepwise changes to current diets by following patterns currently consumed and targeting interventions to specific population groups.

## Data and Code Availability

Data from individual FFQs is protected by the ethical data sharing practices of the Finnish Institute for Health and Welfare (THL). All other data is available along with the code for these analyses at the open repository: https://version.helsinki.fi/rachel.mazac/leg4life

### Supplementary Information

Below is the link to the electronic supplementary material.

Supplementary file1 (DOCX 826 KB)

Supplementary file2 (XLSX 269 KB)

Supplementary file3 (TIF 452 KB)

Supplementary file4 (TIF 159 KB)

Supplementary file5 (PDF 102 KB)

## Footnotes

1 In general, food consumption and usual portion sizes of men and women differ from each other due to e.g., body size and metabolic efficiency. Therefore, in the calculation of food and nutrient intakes based on the FFQ, sex-specific standard portion sizes were applied for most of the 134-items in the FFQ. These standard portion sizes were based on the most recent national food consumption survey [17, 81].

2 See **SM **Table 1, 2, and 3 for the full list of ReCiPe Midpoint (H) impacts and the correlation of each impact with the others using the food groups derived from FFQ, the product LCA processes and list of changes, and the FAO import to export ratio data, respectively.

3 See **SM **Figs. 1 and 2** and SM Table 8** for the PCA results.

4 The individual foods and the groups they are assigned to for the results are listed in **SM **Table 1.

5 For the full list of food ingredients and groups, see **SM **Table 1.

6 For a full list of mBSDS components, nutrient intakes, and food group intakes by cluster, see **SM Table 4**.

7 For the full list and definition of variables, see **SM Table 9**.

8 Further results and calculations can be found **SM Table 6.**

9 See **Supplementary Figs. 1 and 2 and SM Table 8** for the PCA results.

10 Hierarchical clustering results with all variables and clusters can be found in **SM Table 4.**

11 Full results of the contributions of foods consumed and the environmental impact contribution of each food item in each cluster can be found in **SM Table 7.**

12 Calculation of and further results of such diet shifts can be found in **SM Table 6.**

13 Further results of the socioeconomic status, health, lifestyle, and place of habitation for all clusters can be found in **SM Table 5.**

14 We acknowledge, however, that conventional cost–benefit analyses do not capture the potentially catastrophic and systemic risks created by the crossing of planetary boundaries.
