# Correction: Exploring tradeoffs among diet quality and environmental impacts in self-selected diets: a population-based study

**DOI:** 10.1007/s00394-024-03430-x

**Published:** 2024-06-19

**Authors:** Rachel Mazac, Matti Hyyrynen, Niina E. Kaartinen, Satu Männistö, Xavier Irz, Kari Hyytiäinen, Hanna L. Tuomisto, Chiara Lombardini

**Affiliations:** 1 Faculty of Agriculture and Forestry, Department of Agricultural Sciences, Helsinki Institute of Sustainability Science, University of Helsinki, Helsinki, Finland; 2 Natural Resource Institute of Finland, Helsinki, Finland; 3 Finnish Institute for Health and Welfare, Helsinki, Finland; 4 Faculty of Agriculture and Forestry, Department of Economics and Management and Helsinki Institute of Sustainability Science, University of Helsinki, Helsinki, Finland; 5 Faculty of Agriculture and Forestry, Department of Agricultural Sciences, Helsinki Institute of Sustainability Science, Natural Resources Institute Finland (Luke), University of Helsinki, Helsinki, Finland

**Correction to: European Journal of Nutrition**

**DOI:** 10.1007/s00394-024-03366-2

In the original version of this article, in the abstract, second sentence of the result sections which previously read.

One cluster, including twenty percent of the individuals in the sample was identified as a “best compromise” diet with the highest diet quality and the second lowest environmental impacts of all clusters, except for freshwater eutrophication.

Should have read

One cluster, including eighteen percent of the individuals in the sample was identified as a “best compromise” diet with the highest diet quality and the second lowest environmental impacts of all clusters, except for freshwater eutrophication.

