# Supplementary material for: Exploring tradeoffs among diet quality and environmental impacts in self-selected diets: a population-based study
Source: Eur J Nutr. 2024 Apr 7;63(5):1663–78. doi: 10.1007/s00394-024-03366-2 (PMC11329690; doi:10.1007/s00394-024-03366-2)
Supplement: Supplementary file 1 — Supplementary file1 (DOCX 826 KB) [file 394_2024_3366_MOESM1_ESM.docx]

**Exploring tradeoffs among diet quality and environmental impacts in self-selected diets: a population-based study**

Rachel Mazac^1*^, Matti Hyyrynen^2^, Niina E. Kaartinen^3^, Satu Männistö^3^, Xavier Irz^4^, Kari Hyytiäinen^4^, Hanna L. Tuomisto^5^, Chiara Lombardini^4^

^1^University of Helsinki, Faculty of Agriculture and Forestry, Department of Agricultural sciences and Helsinki Institute of Sustainability Science, Helsinki, Finland

^2^Natural Resource Institute of Finland, Helsinki, Finland

^3^Finnish Institute for Health and Welfare, Helsinki, Finland

^4^University of Helsinki, Faculty of Agriculture and Forestry, Department of Economics and Management and Helsinki Institute of Sustainability Science, Helsinki, Finland

^5^University of Helsinki, Faculty of Agriculture and Forestry, Department of Agricultural sciences and Helsinki Institute of Sustainability Science, Helsinki, Finland, Natural Resources Institute Finland (Luke)

*corresponding author: rachel.mazac@helsinki.fi


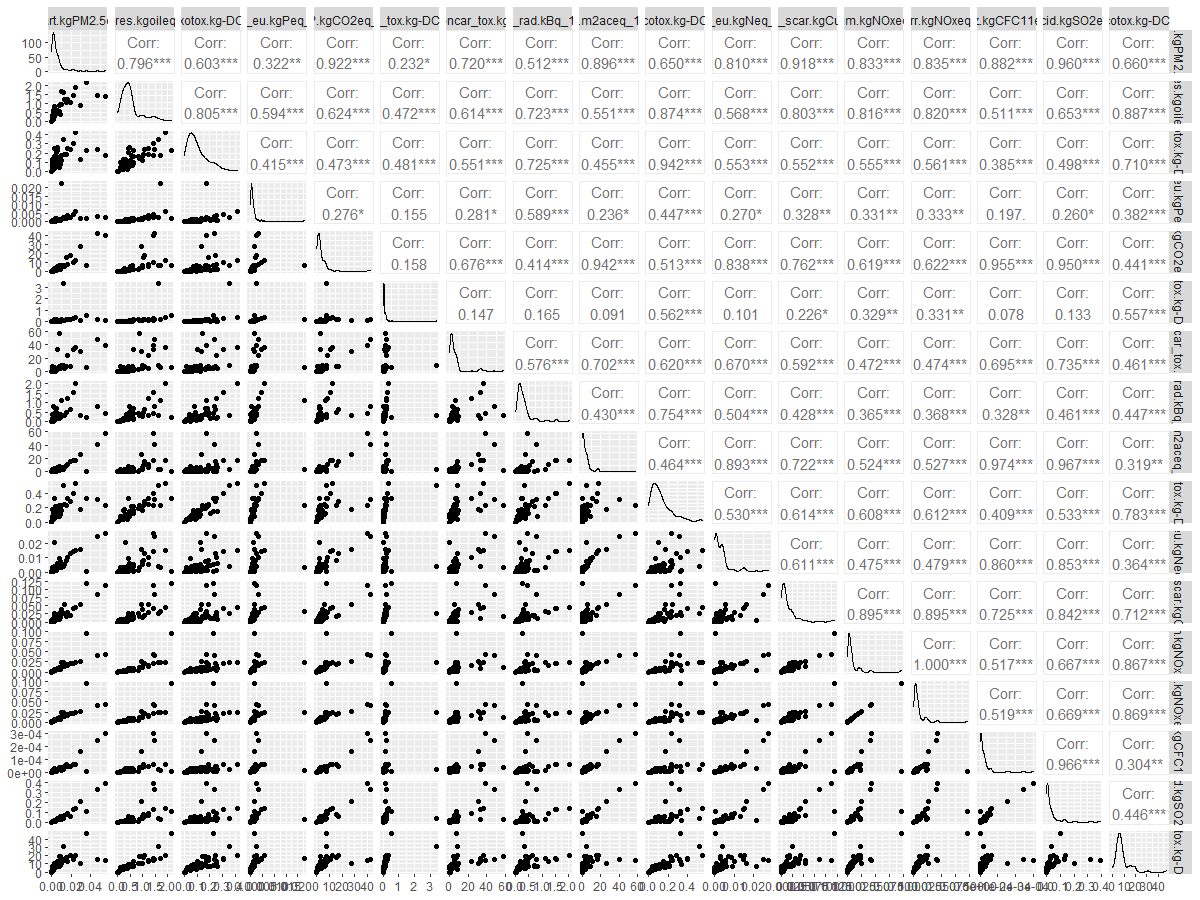


**Supplementary Figure 1.** Correlation coefficients on principal components analysis (PCA) of the 16 environmental impact categories of the ReCiPe 2016 Midpoint (H) Life Cycle Assessment method for the 81 food items included in this study

NOTE: Fine particular matter = fine_part.kgPM2.5eq_1kg

Fossil resource = foss_res.kgoileq_1kg

Freshwater ecotoxicity = frwtr_exotox.kg-DCB_1kg

Freshwater eutrophication = frwtr_eu.kgPeq_1kg

Global warming potential = GWP.kgCO2eq_1kg

Human carcinogenic toxicity = hu_car_tox.kg-DCB_1kg

Human noncarcinogenic toxicity = hu_noncar_tox.kg-DCB

Ironizing radiation = ion_rad.kBq_1kg

Land use (transformation, occupation, relaxation) = LU.m2aceq_1kg

Marine ecotoxicity = mar_ecotox.kg-DCB_1kg

Marine eutrophication = mar_eu.kgNeq_1kg

Mineral resource scarcity = min_res_scar.kgCueq_1kg

Tropospheric ozone formation = oz_hum.kgNOxeq_1kg

Tropospheric ozone (eco) = oz_terr.kgNOxeq_1kg

Stratospheric ozone depletion = strat_oz.kgCFC11eq_1kg

Terrestrial acidification = terr_acid.kgSO2eq_1kg

Terrestrial ecotoxicity = terr_ecotox.kg-DCB_1kg


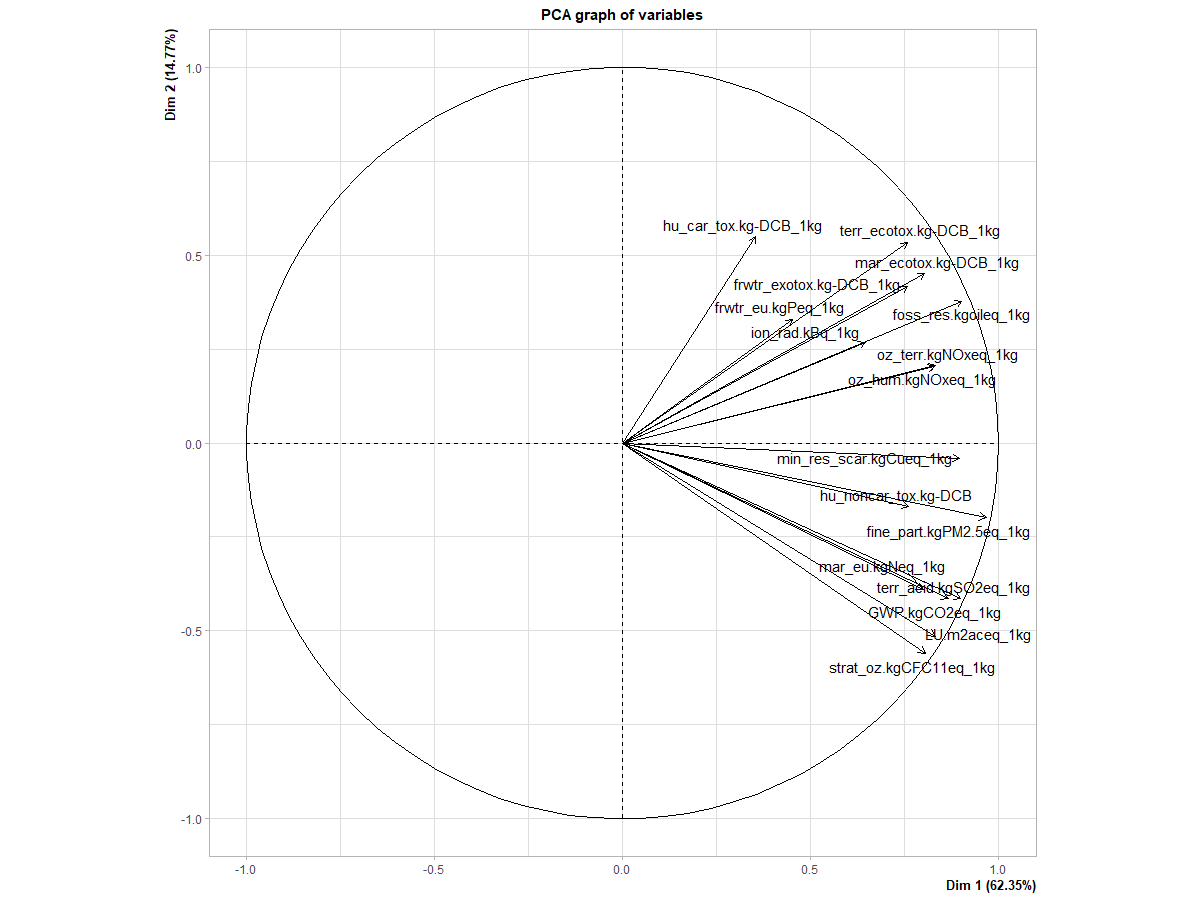


**Supplementary Figure. 2.** Correlation circle of the 16 environmental impact categories (here, variables) of the ReCiPe 2016 Midpoint (H) Life Cycle Assessment method for the 81 food items included in this study.


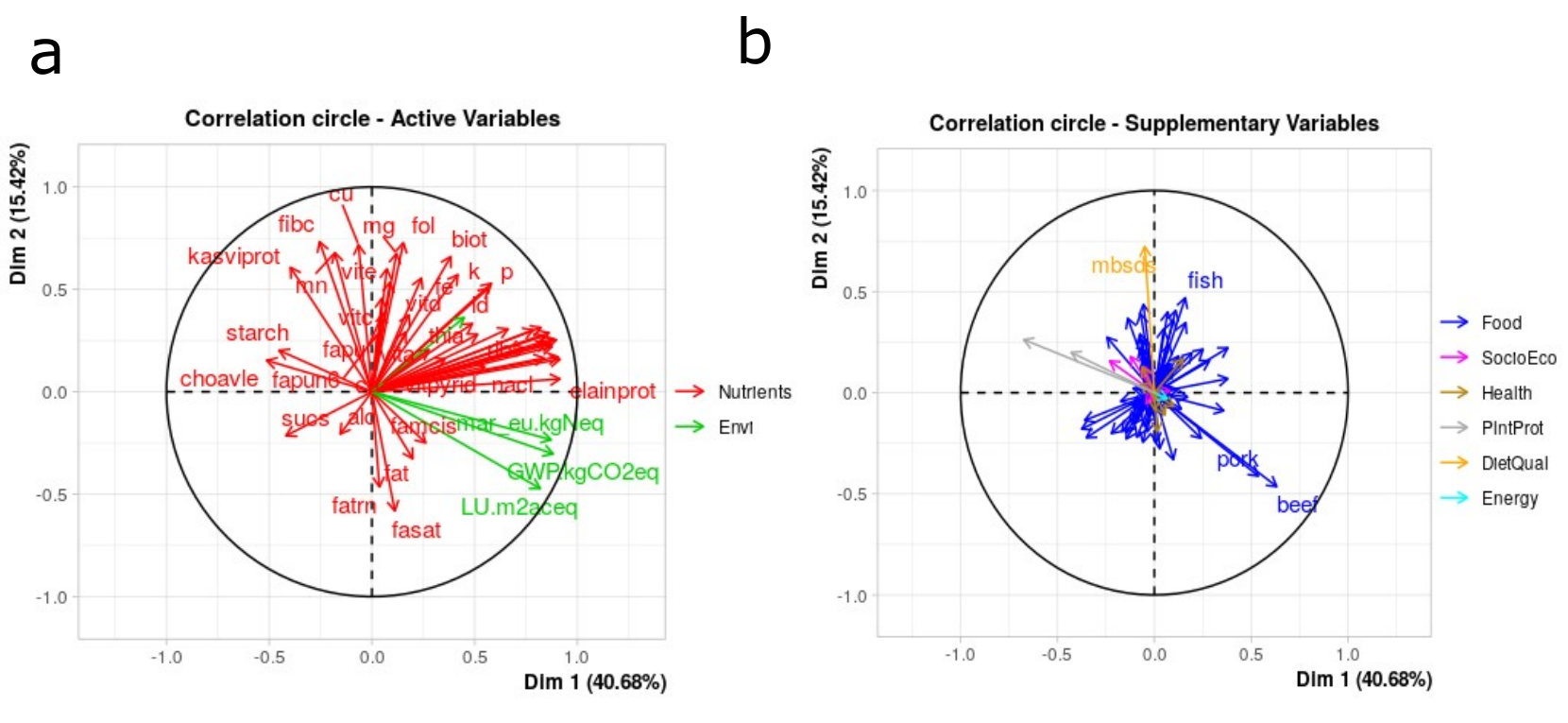


**Supplementary Figure 3**. a. Correlation circle between the active variables and the first two dimensions of the multiple factor analysis (MFA), b. Correlation circle between the supplementary variables and the first two dimensions of the MFA; see list of variables and definitions in **SM Table 9. Food: Food item intakes, SocioEco: Socio-economic variables, Health: health metrics, PlntProt: Plant to animal protein ratios and intake, DietQual: Baltic Sea Diet Score, Energy: total energy intake.**
