# Supplementary figures and images for: Exploring tradeoffs among diet quality and environmental impacts in self-selected diets: a population-based study
Source: Eur J Nutr. 2024 Apr 7;63(5):1663–78. doi: 10.1007/s00394-024-03366-2 (PMC11329690; doi:10.1007/s00394-024-03366-2)

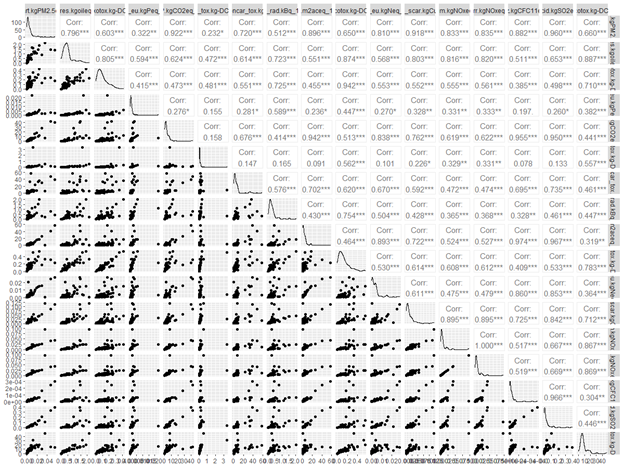

Supplement: Supplementary file 3 — Supplementary file3 (TIF 452 KB) [file 394_2024_3366_MOESM3_ESM.tif]

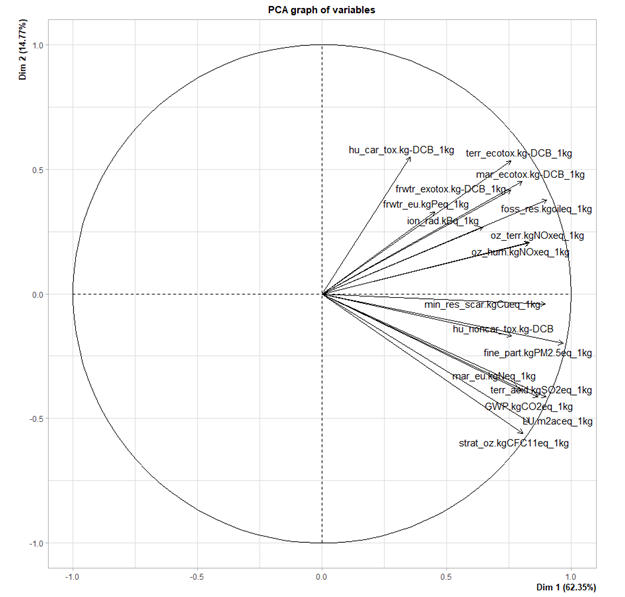

Supplement: Supplementary file 4 — Supplementary file4 (TIF 159 KB) [file 394_2024_3366_MOESM4_ESM.tif]

a

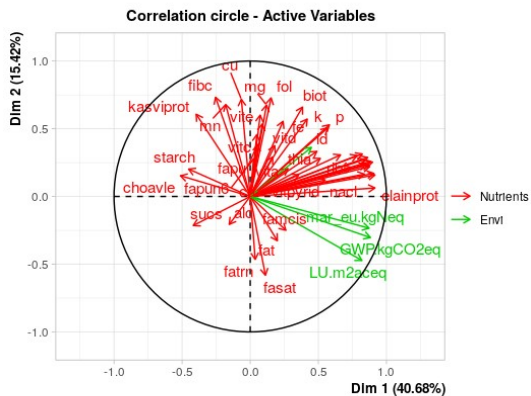

b

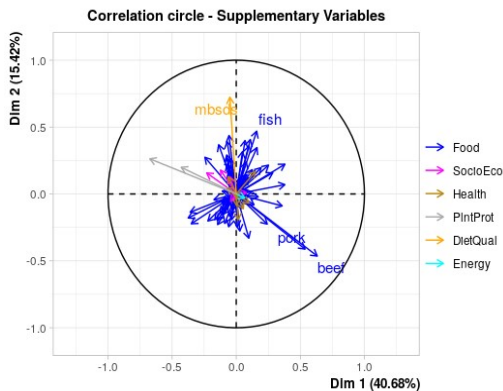

Supplement: Supplementary file 5 — Supplementary file5 (PDF 102 KB) [file 394_2024_3366_MOESM5_ESM.pdf]
